# 

**Published:** 1903-10-24

**Authors:** 


### CONTENTS.

**Research and Post-Graduate Study in Medicine** 59
**Food and Taxation** 60
**Annotations** 61
**Notes and News** 62
**Hospital Clinics and Medical Progress—**
The Treatment of Diabetes 63
Paroxysmal Hæmoglobinuria 64
Gangrene of the Leg following Pneumonia 64
Intra-Cranial Abscess and Middle Ear Disease 65
Gonorrhœal Arthritis and Ophthalmia Neo-Natorum 65
Progress in Opthalmology 66
Progress in Surgery of the Liver and Gall Bladder 67
Progress in State Medicine 63
**New Appliances and Things Medical** 69
**Modern Sociology** 70
**Hospital Administration—**
British Institutions for the Care of the Inebriate.—III. 71
Modern Hospital and Institutional Fittings.—I. 72
The First Garden City 73
**The Student of Medicine** 74

*NURSING SECTION.*

**Notes on News from the Nursing World—**

Workhouse Nursing and the Proposed "Qualified Nurse"—Volunteer Nurses for the Balkans—The Failure of a Nursing Home—Election of Three Matrons—The Age Question—A Nurse for Forty-four Years—Nurses and the Realities of Life—The Leicester Nurses' Home at Norwich—"A Neat, Stylish, and Elegant Matron"—"Supernumerary Assistant Nurses"—Stewardesses with Nursing Training—Nursing at Meerut—Camberwell Guardians and their Matron—Tavistock Cottage Hospital—District Nursing in Kilburn—Making Two Ends Meet at Corwen—Maldon Nursing Association—Our Christmas Distribution—Short Items 51–54

**The Examination of the Urine** 55

**Hints on How to Start a District Nursing Association** 56

**New Books on Nursing** 53

**Everybody's Opinion** 59

**Presentations** 59

**Where to Go** 59

**Appointments** 60

**"The Hospital" Convalescent Fund** 60

**Travel Notes and Queries** 60

**Echoes from the Outside World** 61

**For Reading to the Sick** 62

**Notes and Queries** 62

